# Activation of DNA demethylases attenuates aging‐associated arterial stiffening and hypertension

**DOI:** 10.1111/acel.12762

**Published:** 2018-04-16

**Authors:** Kai Chen, Zhongjie Sun

**Affiliations:** ^1^ Department of Physiology College of Medicine University of Oklahoma Health Sciences Center Oklahoma City OK USA

**Keywords:** aging, AMPK, arterial stiffness, compound H, demethylase, hypertension, secreted Klotho, Sirt1

## Abstract

DNA methylation increases with age. The objective of this study was to investigate whether compound H, a potential activator of DNA demethylases, attenuates aging‐related arterial stiffness and hypertension. Aged mice (24–27 months) and adult mice (12 months) were used. Pulse wave velocity (PWV), a direct measure of arterial stiffness, and blood pressure (BP) were increased significantly in aged mice. Notably, daily treatments with compound H (15 mg/kg, IP) for 2 weeks significantly attenuated the aging‐related increases in PWV and BP. Compound H abolished aging‐associated downregulation of secreted Klotho (SKL) levels in both kidneys and serum likely by enhancing DNA demethylase activity and decreasing DNA methylation. Aging‐related arterial stiffness was associated with accumulation of stiffer collagen and degradation of compliant elastin which are accompanied by increased expression of MMP2, MMP9, TGF‐β1, and TGF‐β3. These changes were effectively attenuated by compound H, suggesting rejuvenation of aged arteries. Compound H also rescued downregulation of Sirt1 deacetylase, AMPKα, and eNOS activities in aortas of aged mice. In cultured smooth muscle cells (SMCc) Klotho‐deficient serum upregulated expression of MMPs and TGFβ which, however, was not affected by compound H. In conclusion, compound H attenuates aging‐associated arterial stiffness and hypertension by activation of DNA demethylase which increases renal SKL expression and consequently circulating SKL levels leading to activation of the Sirt1‐AMPK‐eNOS pathway in aortas of aged mice.

## 1 INTRODUCTION

DNA demethylation is an important process that maintains transcriptional activity of genes (Avrahami et al., 2015; Bigot et al., 2015; Wagner, Fernandez‐Rebollo & Frobel, 2016; Zinovkina & Zinovkin, 2015). An increase in methylation in the promoter region of a gene diminishes the promoter activity and gene transcription (Wagner et al., 2016; Zinovkina & Zinovkin, 2015). Numerous studies showed that DNA methylation is increased with age (Alvares, Mayberry, Joyner, Lakowski & Ahmed, 2014; Avrahami et al., 2015; Bigot et al., 2015; Wagner et al., 2016; Zinovkina & Zinovkin, 2015). Coincidently, the prevalence of arterial stiffness and hypertension also increases with age (Sun, 2015). Arterial stiffening is an independent predictor of cardiovascular outcomes, such as hypertension, myocardial infarction, cognitive decline in aging, stroke, and kidney diseases (Hashimoto & Ito, 2011, 2013; Karras et al., 2012; Kitzman et al., 2013; Sun, 2015). However, the relationship of DNA methylation and aging‐related arterial stiffening is unclear. Whether increased methylation led to arterial stiffening has never been determined. Physiologically, an appropriate methylation level is maintained by the balanced methyltransferase and demethylase activity (Wagner et al., 2016). In this study, we assessed if activation of the demethylase affects arterial stiffening and hypertension in aged mice.

The Klotho gene was originally identified as a putative aging‐suppressor gene in mice that extended lifespan when overexpressed and caused multiple premature aging phenotypes when disrupted (Kuro‐o et al., 1997; Kurosu et al., 2005). The Klotho level decreases with age (Xiao, Zhang, Zheng & Gu, 2004), while the prevalence of arterial stiffness and hypertension increases with age (Kotsis & Stabouli, 2011). At age 70 years, the serum level of Klotho is only about one half of what it was at age 40 years (Xiao et al., 2004). Moreover, the serum Klotho level is significantly decreased in patients with arterial stiffness in chronic kidney diseases (Karras et al., 2012). Our recent study showed that haplodeficiency of Klotho gene caused arterial stiffness (Chen, Zhou & Sun, 2015). We found, in the cultured renal tubule cells, that a small compound (compound H) may be a potential inducer of Klotho gene expression (Jung, Xu & Sun, 2017). Whether compound H promotes Klotho expression and release in vivo has never been determined. In this study, we investigated whether compound H increases Klotho levels and attenuates aging‐associated arterial stiffening and hypertension.

Our results demonstrated that aging‐related arterial stiffening and hypertension are attributed, at least in part, to the increased DNA methylation. Compound H activates demethylases and attenuates arterial stiffening and hypertension in aged mice likely via increasing the Klotho levels.

## 2 METHODS

A detailed method section is available in the Data S1.

### 2.1 Animal study protocols

Six adult mice (12 months) and 20 old mice (24–27 months) were used in this study (129/sv). The old mice were randomly divided into three subgroups and each group had equal number of male mice. Three doses of compound H were tested, and 10 mg/kg bw was chosen as an optimal dose for treating animals. No obvious toxic effect of compound H was observed at this dose. One subgroup received compound H (10 mg kg^−1^ day^−1^, IP, Enamine LLC, Monmouth Jct., NJ) and one group received an equal volume of dimethyl sulfoxide (5%) and served as a control. The structure of compound H was provided in Figure S1. The third group received no treatment. Blood pressure (BP) was measured before and after treatment with compound H at 1 and 2 weeks. Pulse wave velocity (PWV) was measured after 2 weeks treatment with compound H. All animals were euthanized (ketamine/xylazine, 90/10 mg, IP) and perfused transcardially with PBS. The aortas were then quickly removed, washed, and cut into pieces for subsequent analyses.

### 2.2 Histological and immunohistochemical (IHC) examination

The histological and IHC analysis was performed as described in our recent studies (Chen, Lin & Sun, 2016; Gao et al., 2016; Lin, Chen & Sun, 2016; Lin & Sun, 2015a,b; Varshney, Ali, Wu & Sun, 2016). For details, see Data S1.

### 2.3 Western blot analysis

For details, see Data S1.

### 2.4 Reverse transcription–PCR (RT–PCR)

The RT–PCR procedure was performed as described recently (Fan & Sun, 2016; Lin & Sun, 2015b). For details, see Data S1.

### 2.5 Methylation analyses of Klotho gene

For details, see Data S1.

### 2.6 Measurement of DNA demethylase and DNA methyltransferase activity

For details, see Data S1.

### 2.7 Measurement of MMPs activity

For details, see Data S1.

### 2.8 Cell culture and treatment

For details, see Data S1.

### 2.9 Statistical analysis

Quantitative data were presented as the means ± SE. Differences between experimental groups were examined by one‐way analysis of variance (ANOVA) followed by the Bonferroni post‐test using Prism software (GraphPad, La Jolla, CA). For all analysis, *p* < .05 were considered statistically significant.

## 3 RESULTS

### 3.1 Compound H attenuated arterial stiffness and hypertension in aged mice

**Figure 1:**
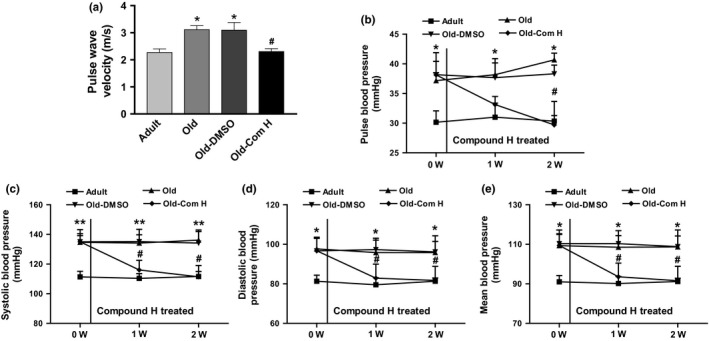
Compound H attenuated arterial stiffness and hypertension in aged mice. (a) PWV was measured by 10‐MHz Doppler probes. (b–e) BP was measured weekly by the volume–pressure recording (VPR) tail‐cuff method using a CODA 6 BP monitoring system during treatment with compound H. Data are expressed as mean ± SE and analyzed by one‐way ANOVA. *n* = 6–7. *p < .05, **p < .01 vs. adult mice; ^#^ *p* < .05, vs. old mice

Pulse wave velocity, a direct measure of arterial stiffness, was increased significantly in aged mice (Figure 1a). The widening of pulse pressure is another direct surrogate of arterial stiffness. Pulse pressure of aged mice was significantly increased compared to that of adult mice (Figure 1b). Thus, aged mice developed arterial stiffness. Daily treatment with compound H for 2 weeks remarkably decreased PWV and pulse pressure in aged mice (Figure 1a,b). Compound H also significantly decreased systolic BP (Figure 1c), diastolic BP (Figure 1d), and mean BP (Figure 1e) in aged mice. Therefore, compound H effectively reduced aging‐related arterial stiffness and hypertension.

### 3.2 Compound H increased secreted Klotho (SKL) expression in aged mice

**Figure 2:**
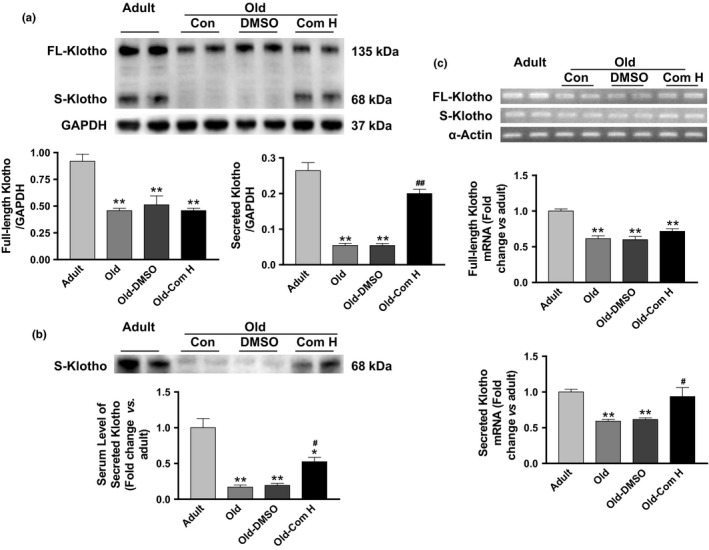
Compound H increased secreted Klotho levels in the kidney and serum of aged mice. (a) Western blot analysis of Klotho expression in kidneys. *n* = 4. (b) Western blot analysis of Klotho in serum (fold change vs. adult). *n *= 4. (c) mRNA expression of Klotho in kidneys (fold change vs. adult). *n *= 4. Data are expressed as mean ± SE and analyzed by one‐way ANOVA. *p < .05, **p < .01 vs. adult mice; ^#^ *p* < .05, ^##^ *p* < .01 vs. old mice

To investigate the potential mechanism contributing to the beneficial effects of compound H on aging‐related arterial stiffening, we measured Klotho expression levels in kidneys, a major source of circulating Klotho (Lindberg et al., 2014; Xu & Sun, 2015b). We found that protein and mRNA expression of both transmembrane (full‐length) and secreted Klotho (SKL) were remarkably decreased in kidneys of aged mice (Figure 2a). Compound H rescued the aging‐related downregulation of SKL expression but did not affect full‐length Klotho expression (Figure 2a). Notably, serum level of SKL was also downregulated in aged mice which was largely rescued by compound H (Figure 2b).

Compound H rescued aging‐associated downregulation of SKL mRNA expression but did not affect full‐length Klotho mRNA in kidneys (Figure 2c).

### 3.3 Compound H attenuated DNA hypermethylation of Klotho gene in aged mice

**Figure 3:**
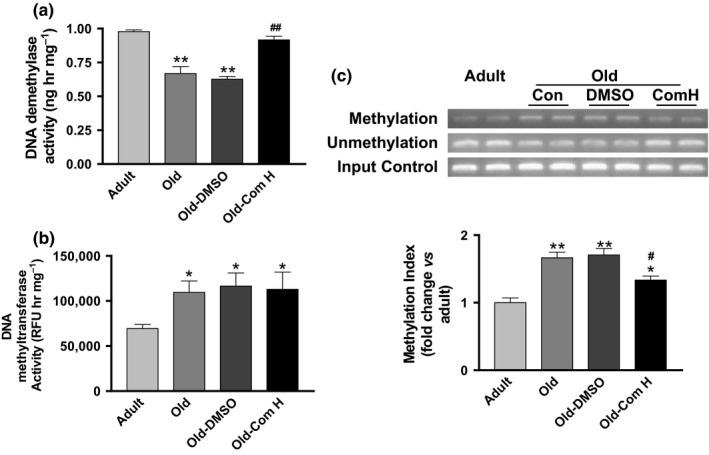
Compound H decreased DNA hypermethylation of Klotho gene in kidneys of aged mice. (a) DNA demethylase activity. *n* = 6. (b) DNA methyltransferase activity. *n* = 6. (c) DNA methylation of Klotho gene (fold change vs. adult). *n* = 4. Data are expressed as mean ± SE and analyzed by one‐way ANOVA. *p < .05, **p < .01 vs. adult mice; ^#^ *p* < .05, ^##^ *p* < .01 vs. old mice

To further explore the potential mechanism of induction of Klotho expression by compound H, we measured the DNA demethylase activity and DNA methyltransferase activity in kidneys. The DNA demethylase activity was decreased while the DNA methyltransferase activity was increased in aged mice (24–27 months old) in relative to those of adult mice (12 months old) (Figure 3a,b). Compound H rescued the downregulation of DNA demethylase activity but did not affect DNA methyltransferase activity in aged mice (Figure 3a,b). We then measured methylation of the Klotho gene. The methylation of CpG islands of the Klotho gene was significantly increased in aged mice compared to that of adult mice (Figure 3c). Compound H effectively attenuated the aging‐related methylation of the Klotho gene (Figure 3c), which was associated with increased expression of SKL in aged mice (Figure 2a). The molecular mechanism of compound H‐induced SKL expression warrants additional investigation.

### 3.4 Compound H attenuated accumulation of stiffer collagen and degeneration of compliant elastin fibers in aortas of aged mice

**Figure 4:**
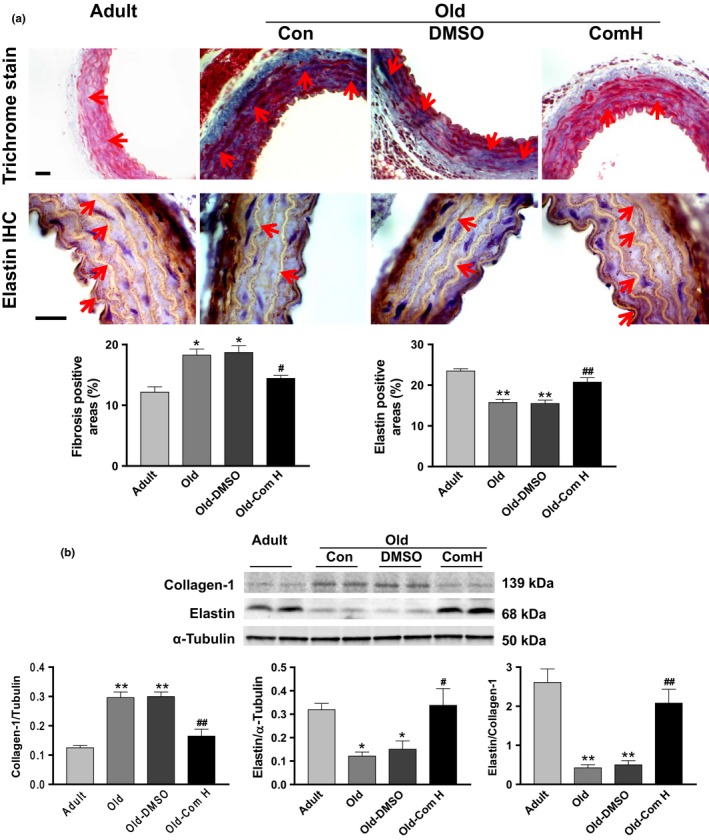
Compound H attenuated the accumulation of collagen and degeneration of elastin in the aorta of aged mice. (a) Histological staining of collagen and immunohistochemical staining of elastin. *n* = 5. (b) Western blot analysis of collagen‐1 and elastin. *n* = 4. Data are expressed as mean ± SE and analyzed by one‐way ANOVA. *p < .05, **p < .01 vs. adult mice; ^#^ *p* < .05, ^##^ *p* < .01 vs. old mice

To investigate the molecular basis of arterial stiffening, we measured arterial collagen and elastin levels. The immunostaining assay showed that aortic collagen levels were increased significantly in aged mice (Figure 4a). The aging‐related collagen deposition (blue) was mainly found in the medial and adventitial layer of the aorta. On the other hand, aortic elastin levels (brown) were decreased significantly in aged mice (Figure 4a). Western blot analysis further confirmed that aging upregulated collagen I levels but downregulated elastin levels in aortas (Figure 4b). The ratio of elastin to collagen in aortas was markedly decreased in aged mice (Figure 4b), indicating that aging causes aortic remodeling. Treatments with compound H abolished upregulation of collagen and downregulation of elastin in aortas leading to attenuation of arterial remodeling in aged mice (Figure 4a,b). No significant differences were found between the Old‐Com H groups and the Adult groups (Figure 4a,b).

### 3.5 Compound H abolished the aging‐associated increases in arterial MMP activity and expression

**Figure 5:**
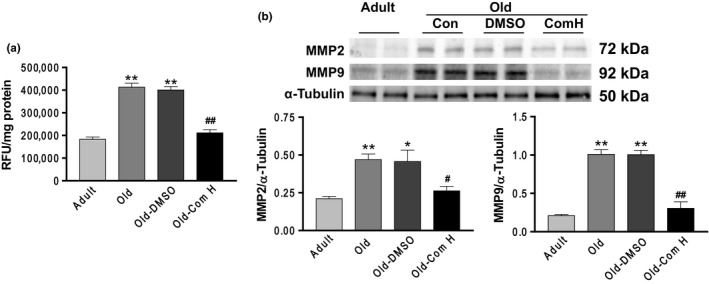
Compound H abolished the aging‐associated increases in arterial MMP activity and expression. (a) MMPs activity analysis. (b) Western blot analysis of MMP2 and MMP9 protein expressions. Data are expressed as mean ± SE and analyzed by one‐way ANOVA. *n *= 4. *p < .05, **p < .01 vs. adult mice; ^#^ *p* < .05, ^##^ *p* < .01 vs. old mice

MMPs are a family of proteases that play important roles in extracellular matrix (ECM) remodeling and degradation. Increased MMP activity could contribute to ECM remodeling and fibrosis. MMPs activity was increased significantly in aortas of aged mice (Figure 5a). An increase in circulating levels of Klotho by compound H remarkably decreased MMPs activity in aged mice (Figure 5a). We also measured MMP protein expression levels by Western blot. MMP2 and MMP9 protein expressions were increased significantly in aortas of aged mice which were effectively attenuated by compound H (Figure 5b). No significant differences in MMPs activity and protein expressions were found between the Old‐Com H groups and the Adult groups (Figure 5a,b).

### 3.6 Aging increased arterial TGF‐β1, TGF‐β3, RUNX2, and ALP expressions which can be abolished by compound H

**Figure 6:**
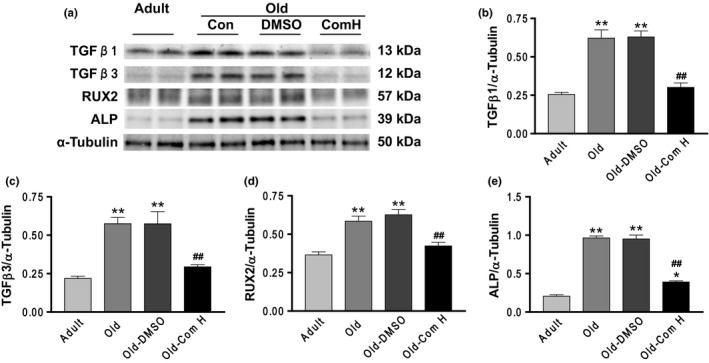
Aging increased arterial TGF‐β1, TGF‐β3, RUNX2, and ALP expressions which can be abolished by compound H. (a) Western blots analysis of TGF‐β1, TGF‐β3, RUNX2, and ALP. (b) Quantification of TGF‐β1 protein levels. (c) Quantification of TGF‐β3 protein levels. (d) Quantification of RUX2 protein levels. (e) Quantification of ALP protein levels. Data are expressed as mean ± SE and analyzed by one‐way ANOVA. *n* = 4. *p < .05, **p < .01 vs. adult mice; ^##^ *p* < .01 vs. old mice

TGFβ promotes matrix protein synthesis and decreases matrix protein degradation, contributing to tissue fibrosis. Runt‐related transcription factor 2 (RUNX2) and alkaline phosphatase (ALP) are markers of fibrosis and arterial stiffening (Capelli, Lusuardi, Cerutti & Donner, 1997; Lin, Chen, Leaf, Speer & Giachelli, 2015). Western blot analysis showed that TGFβ1, TGF‐β3, RUNX2, and ALP expressions were increased significantly in aortas of aged mice (Figure 6a–e). An increase in circulating levels of Klotho by compound H abolished the aging‐related increase in expression of TGFβ1, TGF‐β3, RUNX2, and ALP (Figure 6a–e), suggesting that aging‐associated upregulation of these factors may be due to downregulation of circulating Klotho levels.

### 3.7 Compound H abolished aging‐related downregulation of the SIRT1‐AMPK‐eNOS pathway

Silent information regulator T1 (SIRT1) plays an important role in the regulation of aging and longevity in mammals (Alcendor et al., 2007; Satoh et al., 2013; Wang, Chen, Lv & Liu, 2013). There exist cross‐talks between SIRT1‐ and AMP‐activated protein kinase (AMPK)/eNOS, which are involved in the control of the senescence program (Wang, Liang & Vanhoutte, 2011). Therefore, we assessed the effect of compound H on the SIRT1‐AMPK‐eNOS cascade in aged mice. SIRT1 expression and activity in aortas of aged mice were decreased significantly in relative to the adult mice. Although it did not affect SIRT1 expression, compound H increased SIRT1 activity significantly, as evidenced by an increase in deacetylation of p53 tumor suppressor protein in aged mice (Figure S2A). Consistently, the aging‐related increase in histone acetylation was rescued by compound H (Figure S2A). The phospho‐AMPK and phospho‐eNOS expressions were decreased in the aortas of aged mice (Figure S2B,C), indicating that AMPK and eNOS activities were downregulated with aging. Compound H, an inducer of klotho, rescued the aging‐related downregulation of AMPK and eNOS activities in aortas (Figure S2B,C). Therefore, the aortic SIRT1‐AMPK‐eNOS pathway was suppressed by aging, which can be activated by compound H.

### 3.8 Compound H did not affect MMP2, MMP9, TGFβ1, and TGFβ3 expressions in mouse aortic smooth muscle cells (MOVAS)

The in vivo study showed that compound H increased circulating levels of Klotho and attenuated arterial stiffening and hypertension in aged mice. So we investigated whether compound H and SKL have direct effects on remodeling factors in cultured SMCs. Mouse aortic smooth muscle cells (MOVAS) which do not express endogenous Klotho(Lindberg et al., 2013) were treated with compound H for 16 hr and then harvested for Western blot analysis. In regular medium which has constant levels of Klotho, compound H treatment did not change MMP2, MMP9, TGFβ1, and TGFβ3 expressions (Figure S3), suggesting that compound H did not affect expression of these remodeling factors in SMCs.

In Klotho‐free medium (KL(‐))‐treated cells, MMP2, MMP9, TGFβ1, and TGFβ3 expressions were increased significantly compared to those of regular medium‐treated cells (Figure S3), suggesting that Klotho deficiency directly upregulates these remodeling factors. Notably, SKL protein treatment effectively rescued Klotho deficiency‐induced upregulation of MMP2, MMP9, TGFβ1, and TGFβ3 expressions (Figure S3), suggesting that Klotho directly regulates these factors in SMCs. By contrast, compound H treatment did not affect Klotho deficiency‐induced upregulation of MMP2, MMP9, TGFβ1, and TGFβ3 expressions in SMCs (Figure S3).

These results suggest that the in vivo suppressing effect of compound H on aging‐related upregulation of MMP2, MMP9, TGFβ1, and TGFβ3 expression is mediated by induction of klotho expression and release, which further inhibit arterial remodeling. Therefore, compound H attenuates arterial stiffening and hypertension through increasing circulating levels of Klotho.

## 4 DISCUSSION

SKL may function as a hormone (Xu & Sun, 2015). The circulating SKL may have direct action in vascular endothelial cells or SMCs which do not express SKL. Klotho is an aging‐suppressor gene (Xu & Sun, 2015). Klotho gene deficiency led to arterial stiffening (Chen et al., 2015). This study provides the first evidence that an increase in serum SKL attenuated arterial stiffening.

Aging is associated with increased cardiovascular complications, such as arterial stiffness and hypertension (Sun, 2015). Arterial stiffness is an independent risk factor for myocardial infarction, cognitive decline in aging, stroke, and kidney diseases (Boutouyrie et al., 2002; Matsuoka et al., 2005; Waldstein et al., 2008; Zoungas & Asmar, 2007). Unfortunately, the current antihypertensive drugs were mainly designed to reduce peripheral resistance and are not specific to alter the pathological process of vascular remodeling or stiffening (Dao, Essalihi, Bouvet & Moreau, 2005). Our results showed that PWV and BP were increased in mice of 24–27 months which is equivalent of 70–80 years in humans (Flurkey, Currer & Harrison, 2007), indicating the development of aging‐related arterial stiffness. Importantly, this study demonstrates that activation of demethylases by compound H reversed arterial stiffening and hypertension in aged mice (Figure 1), suggesting that increased methylation plays a critical role in aging‐related arterial stiffening and hypertension. This finding suggests that compound H may be an effective therapeutic agent for arterial stiffness and hypertension.

DNA methylation is one of several epigenetic mechanisms that control gene expression. During development, the pattern of DNA methylation in the genome changes as a result of a dynamic process involving both DNA methylation and demethylation (Moore, Le & Fan, 2013). We measured the methylation state of the promoter region of the Klotho gene to assess whether expression of Klotho, an aging‐suppressor gene, is regulated by DNA methylation in aged mice. We found that aging led to downregulation of DNA demethylase activity and upregulation of DNA methyltransferase activity leading to upregulation of DNA methylation of Klotho gene in aortas of aged mice (Figure 3). Interestingly, compound H rescued the aging‐related downregulation of DNA demethylase activity but did not affect the DNA methyltransferase activity. Compound H restored SKL gene transcription and expression in aged mice (Figure 2) which is likely through attenuating hypermethylation of Klotho gene (Figure 3). Our data suggest that compound H increased DNA demethylase activity in kidneys and increased renal Klotho expression and circulating klotho levels which largely explain the beneficial effect of compound H on arterial stiffening and hypertension. In a recent study, we showed that compound H directly stimulates DNA demethylase activity, decreases DNA methylation, and enhances Klotho expression in the cultured kidney cells (Jung, Xu & Sun, 2017). Our recent study showed that haplodeficiency of Klotho gene caused arterial stiffness and hypertension (Chen et al., 2015). Therefore, we believe that the mechanism of the antihypertensive effect of compound H includes enhanced circulating Klotho levels. The beneficial effect of compound H is mediated, at least partially, by Klotho because compound H does not affect MMPs and TGFβ in vascular SMCs which do not express Klotho (Figure S3). Nevertheless, an additional study is required to determine to what degree the beneficial effect of Compound H is mediated by Klotho.

This study provides the first evidence that stimulation of Klotho expression attenuates aging‐associated arterial stiffening and hypertension. It is intriguing that a small chemical compound effectively induced SKL expression and increases circulating levels of SKL. It was reported that at age of 70 years, the serum level of Klotho is only about one half of what it was at age of 40 years (Xiao et al., 2004), while the prevalence of arterial stiffness and hypertension is increased in the aged population (Kotsis & Stabouli, 2011). Thus, Klotho deficiency may be a pathological factor for aging‐associated arterial stiffness and hypertension. It is interesting that a kidney‐derived protein (SKL) plays a critical role in the maintenance of normal vascular structure and function.

We further investigated whether compound H and SKL affect remodeling factors in MOVAS which do not express endogenous Klotho. Compound H had no direct effect on expression of MMP2, MMP9, TGFβ1, and TGFβ3 in cultured MOVAS (Figure S3). By contrast, SKL‐deficient medium upregulated MMP2, MMP9, TGFβ1, and TGFβ3 expressions in MOVAS. Interestingly, Klotho deficiency‐induced upregulation of these factors was abolished by adding SKL but was not affected by compound H. Compound H may not affect demethylase or methyltransferase activities in vascular cells because it did not alter expression of MMPs and TGFβ in cultured SMCs (Figure S3). This hypothesis needs to be validated.

It is noticed that compound H reversed aging‐associated arterial stiffening and remodeling, suggesting that compound H *via* induction of SKL expression and release rejuvenates aged arteries. The beneficial effect of compound H may be partially attributed to reversal of upregulation of collagen and downregulation of elastin (Figure 4). This process may include collagen degradation and elastin synthesis which may be likely due to upregulation of MMP and TGFβ induced by increased SKL levels. Although aging led to a decrease in autophagy, the beneficial effect of compound H may not involve autophagy which was not affected by compound H (Figure S4).

Two types of Klotho protein with potentially different functions have been identified: a full‐length transmembrane Klotho and a SKL (Xu & Sun, 2015). The full‐length Klotho is mainly expressed in kidney distal tubule cells and serves as a coreceptor of FGF23 and enhances FGF23 signaling to maintain phosphate homeostasis. The circulating Klotho, that is SKL, may act as a hormone and regulate the functions of tissues or cells that do not express Klotho (e.g., vascular endothelial cells and smooth muscle cells) (Lindberg et al., 2013; Xu & Sun, 2015b). In this study, we found that both full‐length and SKL in kidneys were decreased in aged mice (Figure 2). However, compound H only rescued the downregulation of SKL but did not affect transmembrane Klotho. The molecular mechanism of the selective action of compound H on SKL remains to be explored. SKL and the full‐length klotho are encoded by the same gene. SKL was generated due to alternative splicing (Xu & Sun, 2015). Thus, compound H might demethylase an alternative RNA splicer to rescue the aging‐related downregulation of SKL. This hypothesis warrants further investigation.

SIRT1, known as a class III histone deacetylase, is a nuclear protein implicated in the regulation of many cellular processes, including apoptosis, cellular senescence, endocrine signaling, glucose homeostasis, aging, and longevity. SIRT1 has been reported to deacetylate the lysine residues of a number of nuclear proteins, such as p53 (Yuan et al., 2011), NF‐κB (Salminen & Kaarniranta, 2009), PGC‐1a (Amat et al., 2009), CBP/p300 (Das, Lucia, Hansen & Tyler, 2009), and forkhead family proteins (Brunet et al., 2004). Several recent studies demonstrated that Sirt1 could inhibit TGF‐β signaling and ameliorate fibrosis (Huang et al., 2014; Kume et al., 2007; Zerr et al., 2014). In this study, we found that aging suppressed SIRT1 activity, increased TGFβ and MMP expressions, and induced fibrosis. Compound H rescued aging‐associated downregulation of SIRT1 expression in aortas likely by increasing the circulating SKL levels. Haplodeficiency of Klotho gene led to downregulation of vascular SIRT1 (Gao et al., 2016). Pharmacological activation of SIRT1 attenuated Klotho deficiency‐induced arterial stiffening (Gao et al., 2016). Therefore, downregulation of SIRT1 activity may be an important factor contributing to aging‐induced arterial remodeling and stiffness.

However, the recent studies showed that large‐artery stiffness precedes the development of hypertension (Kaess et al., 2012; Najjar et al., 2008). Although hypertension could also contribute to vascular remodeling and stiffening, it is, however, a slow process. A reduction in BP by 20 mmHg may be insufficient to reverse aging‐related arterial remodeling or arterial stiffening in 2 weeks. Nevertheless, this study does not exclude the possibility that attenuation of BP by compound H may also contribute to the improvement of arterial stiffening in aging mice. The limitation of this study is that it does not elucidate the relationship of arterial stiffening and hypertension (causality). It is noticed that compound H decreased aging‐associated arterial stiffening and hypertension within 2 weeks, which is sooner than expected. This finding suggests that there may be a functional component in arterial stiffening, that is, increased vascular tone. The acute effect of compound H may be partially due to improved endothelial function and decreased vascular resistance. Indeed, treatment with compound H, via stimulation of SKL release, activated the SIRT1‐AMPKα‐eNOS pathway as evidenced by increased activities of SIRT1, AMPKα, and eNOS (Figure S2A–C). eNOS is an important enzyme in the regulation of BP, vascular tone, and angiogenesis. Several protein kinases including AMPK activate eNOS by phosphorylating Ser1177 in response to various stimuli (Dimmeler et al., 1999; Fulton et al., 1999). Therefore, the mechanism of the antihypertensive effect of compound H may include activation of the SIRT1‐AMPK‐eNOS pathway, inhibition of MMP and TGFβ, and attenuation of arterial remodeling and stiffening.

## 5 PERSPECTIVE

Our study provides the first experimental evidence that aging‐associated arterial stiffening and hypertension are attributed, at least in part, to increased DNA methylation. It is new and interesting that compound H activates DNA demethylase which induces Klotho expression and release leading to attenuation of arterial stiffening and hypertension in aged mice. Our study also provides the first evidence that compound H may be an effective therapeutic agent for aging‐related arterial stiffening and hypertension.

## CONFLICT OF INTEREST

None declared.

## AUTHORS' CONTRIBUTION

ZS, design experiment, critical editing manuscript; KC, perform experiment, collect data, and write manuscript.
