# Supplementary material for: Activation of DNA demethylases attenuates aging‐associated arterial stiffening and hypertension
Source: Aging Cell. 2018 Apr 16;17(4):e12762. doi: 10.1111/acel.12762 (PMC6052484; doi:10.1111/acel.12762)
Supplement: Supplementary file 1 [file ACEL-17-na-s001.docx]

**Online Supplemental Methods and Data**

**Activation of DNA Demethylases Attenuates Aging-associated Arterial Stiffening and Hypertension**

Kai Chen, Zhongjie Sun

Department of Physiology, College of Medicine, University of Oklahoma Health Sciences Center, Oklahoma City, OK73104, USA

**Running Title:** Compound H & Aging Hypertension

Address Correspondence to:

Zhongjie Sun, MD, PhD, FAHA

Chair, Research Committee

Professor of Physiology

Director, The Robert & Mary Cade Laboratory

BMSB 662A, Box 26901

Department of Physiology, BMSB 662A

College of Medicine

University of Oklahoma Health Sciences Center (OUHSC)

940 Stanton L. Young Blvd.

Oklahoma City, OK 73126-0901

USA

[Zhongjie-Sun@ouhsc.edu](mailto:Zhongjie-Sun@ouhsc.edu)

Tel. 405-271-2226 x56237

Fax. 405-271-318

**Materials and Methods**

**Animal Study Protocols**

This study was performed according to the guidelines of the National Institute of Health on the care and use of laboratory animals and approved by the Institutional Animal Care and Use Committee of University of Oklahoma Health Science Center. All mice were housed in cages at room temperatures (25±1°C) and were provided with Purina laboratory chow (No. 5001) and tap water ad libitum.

**Cell Culture and Treatment**

MOVAS (ATCC^®CRL-2797^) is a continuous mouse aortic vascular smooth muscle cell line that has been demonstrated to retain a VSMC-like phenotype, including a spindle cell morphology and the expression of VSMC-specific markers such as smooth muscle α-actin and SM22-α. MOVAS were cultured in Dulbecco's modified Eagle's medium (DMEM) supplemented with 10% heat-inactivated fetal bovine serum (FBS) and 2mM L-glutamine. After cells confluent, the media were switched to DMEM with or without 2% serum, and the cells were then treated with 10 μM compound H or 5 nM secreted Klotho for another 16 h before harvest for western blot analysis.

**Measurement of Pulse Wave Velocity**

Aortic PWV was measured as described previously ([Hartley, Taffet, Michael, Pham, & Entman, 1997](#_ENREF_5); [Reddy et al., 2003](#_ENREF_7)). Briefly, mice were anesthetized under 2% isoflurane in the SomnoSuite anesthesia system (Kent Scientific, Torrington, CT) for 1~3 min. Anesthesia was maintained *via* nose cone, and mice were secured in a supine position on a heating board (~37°C) to maintain body temperature. Velocity signals from transverse aortic arch and abdominal aorta were obtained using a 10-MHz pulsed Doppler probes (Indus Instruments, Webster, TX) and collected using Doppler signal processing workstation. Absolute pulse arrival times were indicated by the sharp upstroke of each velocity waveform. Aortic PWV was then calculated as the quotient of the separation distance, assessed to the nearest half millimeter by engineering caliper, and difference in absolute arrival times.

**Measurement of Blood pressure**

Blood pressure was measured by a computerized volume-pressure recording (VPR) tail-cuff method with slight warming (28°C) but not heating of the tail using a CODA-6 none-invasive blood pressure monitoring system (Kent Scientific, Torrington, CT) as we described previously ([Crosswhite & Sun, 2010](#_ENREF_1); [Lin, Chen, & Sun, 2016](#_ENREF_6)). This method has been validated by others using a telemetry system ([Feng et al., 2008](#_ENREF_3); [Whitesall, Hoff, Vollmer, & D'Alecy, 2004](#_ENREF_12)). Animals were gently handled and trained for the VPR tail-cuff measurement to minimize handling stress. No signs of stress were observed during BP measurements. The operator was also strictly trained for the measurement procedure. At least 20 stable cycle data were obtained for the result analysis for each measurement. The VPR method also has been validated in our lab by direct arterial cannulation ([Gao et al., 2016](#_ENREF_4)) and by telemetry ([X. Wang & Sun, 2010](#_ENREF_10)). The VPR method can reliably monitor BP and is a common method for monitoring BP in our laboratory ([Gao et al., 2016](#_ENREF_4); [Lin et al., 2016](#_ENREF_6); [X. Wang & Sun, 2010](#_ENREF_10); [Y. Wang & Sun, 2009](#_ENREF_11)).

**Histological and immunohistochemical examination**

### Thoracic aortas were quickly excised and placed in cold (4°C) physiological saline solution. Three millimeter rings with perivascular tissue intact were removed from the thoracic aorta directly distal to the greater curvature of the aortic arch. Aorta rings were post-fixed in 4% paraformaldehyde, embedded in paraffin and sectioned at 5 μm thickness. Collagen was revealed using masson’s trichrome staining as described previously ([X. Wang et al., 2012](#_ENREF_9)). The blue staining represented collagen deposition. A series of 6-8 sections of each mouse (5 mice per group) were examined and photographed using an Olympus BH-L microscope coupled with a digital color camera. Blue-stained collagen areas were quantified with ImageJ (NIH, Bethesda, MA) from 4-5 regions per section. The same threshold was used for each photo to make sure they are comparable.

### Elastin was assessed by immunohistochemical visualization. Briefly, sections are washed and incubated in primary antibody against elastin (1:50, Abcam, Cambridge, MA, USA) or negative control (2.5% horse serum) overnight. Detection was performed using an HRP-conjugated secondary antibody followed by chromogenic detection using DAB as the substrate. Finally, series of 6-8 sections were examined and photographed using an Olympus BH-L microscope coupled with a digital color camera. Elastin levels were quantified with ImageJ (NIH, Bethesda, MA) from 4-5 regions per section. The same threshold was used for each photo to make sure they are comparable.

**Collagen and elastin extraction**

Collagen and elastin are very insoluble proteins complex that are difficult to extract. So the extraction was performed with pepsin at the concentration of 0.1 mg/ ml in 0.5M acetic acid at 4 °C for 12 h ([Faris et al., 1978](#_ENREF_2)). In detail, thoracic aorta were cut into small pieces and treated with RIPA buffer. The supernatant were kept for measuring soluble proteins by western blot. The pellets were then added to 0.1 mg/ ml pepsin in 0.5M acetic acid at 4 °C for 12 h with stirring. Then centrifuge extract for 10 minutes at 14,000 x g in a cold microfuge and remove supernatant for collagen and elastin measurement.

**Western Blot Analysis**

The proteins (40–50 mg) were resolved by SDS-PAGE and transferred to a nitrocellulose membrane (Bio-Rad, [Hercules, CA](https://www.google.com/search?q=Hercules+California&stick=H4sIAAAAAAAAAOPgE-LSz9U3MC4wzDVPUeIAsQsrCwu1tLKTrfTzi9IT8zKrEksy8_NQOFYZqYkphaWJRSWpRcUALCJywkQAAAA&sa=X&ved=0ahUKEwj89eWE_cDXAhUP7GMKHUnpCxAQmxMInwEoATAS)). The membrane was then incubated overnight (4°C) with a primary antibody against Klotho (R&D, 1:200), collagen-1, elastin, MMP2, MMP9, ALP (Abcam, 1:1000), TGFβ-1, TGFβ-3 (Santa Cruz, 1:100), RUX2, or α-Tubulin (Cell Signaling, 1:1000). The rabbit anti-goat ,goat anti-mouse or goat anti-rabbit horseradish peroxidase (1:2000–1:5,000; Santa Cruz) was used as a secondary antibody and incubated for 1 hour at room temperature. Specific proteins were detected by chemiluminescent methods using Clarity western ECL substrate (Bio-Rad, [Hercules, CA](https://www.google.com/search?q=Hercules+California&stick=H4sIAAAAAAAAAOPgE-LSz9U3MC4wzDVPUeIAsQsrCwu1tLKTrfTzi9IT8zKrEksy8_NQOFYZqYkphaWJRSWpRcUALCJywkQAAAA&sa=X&ved=0ahUKEwj89eWE_cDXAhUP7GMKHUnpCxAQmxMInwEoATAS)). Protein abundance on western blots was quantified by densitometry using Image lab software (Bio-Rad, Hercules, CA).

**Reverse transcription-PCR (RT-PCR)**

Total RNA was extracted using a Direct-zol ^TM^ RNA Miniprep kit (Zymo Research, Irvine, CA) from kidney of adult and aged mice. The first-strand cDNA was synthesized from 500 ng of total RNA by using an iScript cDNA synthesis kit (Bio-Rad, Hercules, CA). The PCR conditions for all primers were as follows: hold for 5 min at 94 °C, followed by 30 cycles consisting of denaturation at 94 °C (30 s), annealing at 57 °C (30 s), and elongation at 72 °C (1 min). The amplified products were subjected to electrophoresis on a 1% agarose gel. Each reaction was performed in triplicates. The gene expression was calculated as band intensity of Klotho / band intensity of α-Actin and plotted after normalization to the control group. The primers for Klotho were provided in Online Supplemental Table S1.

**Methylation analyses of Klotho gene**

The methylation status of CpG islands of the mouse Klotho gene was analyzed using methylation-specific PCR (MSP) and bisulfite sequencing ([Sun, Chang, & Wu, 2012](#_ENREF_8)). First, the genomic DNA was extracted from kidneys of adult and aged mice with tissue DNA Kit (Omega, Norlross, GA) and modified by bisulfate treatment (EZ DNA Methylation-Gold Kit, Zymo Research, Irvine, CA) for MSP analyses with gene promoter–specific primer pair that recognize the methylated and unmethylated CpG sites. The PCR product of genomic DNA without bisulfate treatment, with primers located in the promoter region of the mouse Klotho gene, was used as the inputted control for the MSP. The PCR products were visualized by ethidium bromide staining in 2% agarose gels, and the densitometric intensity corresponding to each band was quantified. Each reaction was performed in triplicates. The methylation index was calculated as (band intensity of MSP with methylated primers) / (band intensity of inputted control). The methylation index was plotted after normalization to the control group. The sequence of MSP primers and the amplification program were summarized in Online Supplemental Table S2 online.

**Measurement of DNA demethylase and DNA methyltransferase activity**

The DNA demethylase and DNA methyltransferase activity were measured by using DNA demethylase activity qualification kit (Abcam, Cambridge, MA) and methyltransferase colorimetric assay kit (Cayman, Ann Arbor, Michigan). Protein extracts from the thoracic aorta were prepared in lysis buffer. Assay procedures were followed by the manufacturer’s protocols. The absorbance was read at 450 nm for DNA demethylase activity assay and 510 nm for DNA methyltransferase activity using the Synergy 2 multi-mode reader (BioTek, Winooki, VT).

**Measurement of MMPs activity**

MMPs activity were measured using MMP activity assay kit (Abcam, Cambridge, MA). Briefly, Lysates from thoracic aorta were quantified using Pierce BCA assay (Thermo Fisher, [Waltham, MA](https://www.google.com/search?q=Waltham+Massachusetts&stick=H4sIAAAAAAAAAOPgE-LSz9U3MCooMTBJU-IAsTOqjE21tLKTrfTzi9IT8zKrEksy8_NQOFYZqYkphaWJRSWpRcUAAxikqkQAAAA&sa=X&ved=0ahUKEwiu2M-a_8DXAhXJ3YMKHUulAC0QmxMIoAEoATAV)) and prepared under non-reducing, non-denaturing conditions. The MMP containing-samples were incubated with equal volume of 2 mM APMA working solution for 10-15 minutes to activate pro-MMPs. 50 μL of MMP Green Substrate working solutions were then added to the sample and control wells of the assay plate and incubated for 30 minutes. The fluorescence intensity were monitored with a fluorescence plate reader at Ex/Em = 490/525 nm. The MMPs activity were calculated by relative fluorescence units (RFU) normalized by proteins quantification.

**References**

Crosswhite, P., & Sun, Z. (2010). Ribonucleic acid interference knockdown of interleukin 6 attenuates cold-induced hypertension. *Hypertension, 55*(6), 1484-1491. doi:HYPERTENSIONAHA.109.146902 [pii]

10.1161/HYPERTENSIONAHA.109.146902 [doi]

Faris, B., Moscaritolo, R., Levine, A., Snider, R., Goldstein, R., & Franzblau, C. (1978). Isolation of purified insoluble aortic collagen. *Biochim Biophys Acta, 534*(1), 64-72. Retrieved from <http://www.ncbi.nlm.nih.gov/pubmed/656467>

Feng, M., Whitesall, S., Zhang, Y., Beibel, M., D'Alecy, L., & DiPetrillo, K. (2008). Validation of volume-pressure recording tail-cuff blood pressure measurements. *Am J Hypertens, 21*(12), 1288-1291. Retrieved from <http://www.ncbi.nlm.nih.gov/entrez/query.fcgi?cmd=Retrieve&db=PubMed&dopt=Citation&list_uids=18846043>

Gao, D., Zuo, Z., Tian, J., Ali, Q., Lin, Y., Lei, H., & Sun, Z. (2016). Activation of SIRT1 Attenuates Klotho Deficiency-Induced Arterial Stiffness and Hypertension by Enhancing AMP-Activated Protein Kinase Activity. *Hypertension, 68*(5), 1191-1199. doi:10.1161/hypertensionaha.116.07709

Hartley, C. J., Taffet, G. E., Michael, L. H., Pham, T. T., & Entman, M. L. (1997). Noninvasive determination of pulse-wave velocity in mice. *Am J Physiol, 273*(1 Pt 2), H494-500. Retrieved from <http://www.ncbi.nlm.nih.gov/pubmed/9249523>

Lin, Y., Chen, J., & Sun, Z. (2016). Antiaging Gene Klotho Deficiency Promoted High-Fat Diet-Induced Arterial Stiffening via Inactivation of AMP-Activated Protein Kinase. *Hypertension, 67*(3), 564-573. doi:HYPERTENSIONAHA.115.06825 [pii]

10.1161/HYPERTENSIONAHA.115.06825 [doi]

Reddy, A. K., Li, Y. H., Pham, T. T., Ochoa, L. N., Trevino, M. T., Hartley, C. J., . . . Taffet, G. E. (2003). Measurement of aortic input impedance in mice: effects of age on aortic stiffness. *Am J Physiol Heart Circ Physiol, 285*(4), H1464-1470. doi:10.1152/ajpheart.00004.2003 [doi]

00004.2003 [pii]

Sun, C. Y., Chang, S. C., & Wu, M. S. (2012). Suppression of Klotho expression by protein-bound uremic toxins is associated with increased DNA methyltransferase expression and DNA hypermethylation. *Kidney Int, 81*(7), 640-650. doi:10.1038/ki.2011.445

Wang, X., Skelley, L., Wang, B., Mejia, A., Sapozhnikov, V., & Sun, Z. (2012). AAV-Based RNAi Silencing of NADPH Oxidase gp91(phox) Attenuates Cold-Induced Cardiovascular Dysfunction. *Human Gene Therapy, 23*(9), 1016-1026.

Wang, X., & Sun, Z. (2010). RNAi silencing of brain klotho potentiates cold-induced elevation of blood pressure via the endothelin pathway. *Physiol Genomics, 41*, 120-126. doi:00192.2009 [pii]

10.1152/physiolgenomics.00192.2009 [doi]

Wang, Y., & Sun, Z. (2009). Klotho gene delivery prevents the progression of spontaneous hypertension and renal damage. *Hypertension, 54*(4), 810-817. doi:HYPERTENSIONAHA.109.134320 [pii]

10.1161/HYPERTENSIONAHA.109.134320 [doi]

Whitesall, S. E., Hoff, J. B., Vollmer, A. P., & D'Alecy, L. G. (2004). Comparison of simultaneous measurement of mouse systolic arterial blood pressure by radiotelemetry and tail-cuff methods. *Am J Physiol Heart Circ Physiol, 286*(6), H2408-2415. Retrieved from <http://www.ncbi.nlm.nih.gov/entrez/query.fcgi?cmd=Retrieve&db=PubMed&dopt=Citation&list_uids=14962829>

**Online Supplemental Tables**

**Supplemental Table S1. The sequence of Klotho and α-Actin primers**

|  | **Forward, 5'-3'** | **Backward, 5'-3'** |
| --- | --- | --- |
| Full-length Klotho | ACGTTCAAGTGGACACTACTCT | TTCTTGGCTACAACCCCGTC |
| Secreted Klotho | CCTGTGACTTTGCTTGGGGA | GTTTGGCTCAACGTCCCAAC |
| α-Actin | GTCGAGTCGCGTCCACC | GTCATCCATGGCGAACTGGT |

**Supplemental Table S2. The sequence of MSP primers and the amplification program**

|  | **Forward, 5'-3'** | | **Backward, 5'-3'** |
| --- | --- | --- | --- |
| Klotho set |  | |  |
| Methylated | CGTTGTTTGAGCGTTGAGTC | | AAACCGTCGAAAAAAATATCGTA |
| Unmethylated | TTTGTTGTTTGAGTGTTGAGTTG | | AAAAACCATCAAAAAAAATATCATA |
| Inputted control (Klotho) | TAGTTTTAGGAAGGTAAAGGGAGTG | | AAATACCCAAAAAAAACACAACAAA |
|  | | **Program** | |
| Klotho set | | 95。C x 8 min;( 95。C x 1min, 61。C x 1min, 72。C x 2.5 min) x 40 cycles; 72。C 5 min | |
| Inputted control (Klotho) | | 95。C x 8 min;( 95。C x 1min, 59。C x 1min, 72。C x 2.5 min) x 40 cycles; 72。C 5 min | |

**Online Supplemental Figures**

**Supplemental Figure S1.** The structure of compound H.

**Supplemental Figure S2. Compound H abolished aging-associated downregulation of the SIRT1-AMPK-eNOS pathway. (A)** Western blots analysis of SirT1, and it’s substrate p53. **(B)** Western blots analysis of P-AMPK and AMPK. **(C)** Western blots analysis of P-eNOS and eNOS (fold change *vs.* adult). Data are expressed as mean ± SE and analyzed by one-way ANOVA. n=4. *p<0.05, **p<0.01 *vs.* adult mice; #p<0.05, ^##^p<0.01 *vs.* old mice.

**Supplemental Figure S3. Compound H did not affect MMP2, MMP9, TGFβ1, and TGFβ3 expression in mouse vascular aortic smooth muscle cells (MOVAS).** MOVAS were treated with compound H, Klotho free (KL (-)) medium, and/or secreted Klotho (SKL) for 16 h and then harvested for western blot analysis. Data are expressed as mean ± SE and analyzed by one-way ANOVA. n=4. *p<0.05, **p<0.01 *vs.* regular medium; #p<0.05, ^##^p<0.01 *vs.* KL (-) medium.

**Supplemental Figure S4. Compound H did not affect autophagy expression in aorta (A), heart (B), and kidney (C) in aged mice.** Data are expressed as mean ± SE and analyzed by one-way ANOVA. n=4. *p<0.05, **p<0.01 *vs*. adult mice.
